# Immunizing Mice with Influenza Virus-like Particles Expressing the *Leishmania amazonensis* Promastigote Surface Antigen Alleviates Inflammation in Footpad

**DOI:** 10.3390/vaccines12070793

**Published:** 2024-07-18

**Authors:** Gi-Deok Eom, Ki Back Chu, Keon-Woong Yoon, Jie Mao, Sung Soo Kim, Fu-Shi Quan

**Affiliations:** 1 Department of Biomedical Science, Graduate School, Kyung Hee University, Seoul 02447, Republic of Korea; ekd3910@naver.com (G.-D.E.); kgang92@gmail.com (K.-W.Y.); maojie@khu.ac.kr (J.M.); 2 Department of Parasitology, Inje University College of Medicine, Busan 47392, Republic of Korea; ckb421@gmail.com; 3 Department of Infectious Disease and Malaria, Paik Institute of Clinical Research, Inje University, Busan 47392, Republic of Korea; 4 Medical Research Center for Bioreaction to Reactive Oxygen Species and Biomedical Science Institute, Core Research Institute (CRI), Kyung Hee University, Seoul 02447, Republic of Korea; sgskim@khu.ac.kr; 5 Department of Medical Zoology, School of Medicine, Kyung Hee University, Seoul 02447, Republic of Korea

**Keywords:** *Leishmania amazonensis*, promastigote surface antigen, virus-like particle, vaccine

## Abstract

Cutaneous leishmaniasis (CL) is a tropical disease endemic in many parts of the world. Characteristic clinical manifestations of CL include the formation of ulcerative skin lesions that can inflict life-long disability if left untreated. Although drugs are available, they are unaffordable and out of reach for individuals who need them the most. Developing a highly cost-efficient CL vaccine could address this problem but such a vaccine remains unavailable. Here, we developed a chimeric influenza virus-like particle expressing the *Leishmania amazonensis* promastigote surface antigen (LaPSA-VLP). LaPSA-VLPs were self-assembled in *Spodoptera frugiperda* insect cell lines using the baculovirus expression system. After characterizing the vaccines and confirming successful VLP assembly, BALB/c mice were immunized with these vaccines for efficacy assessment. Sera acquired from mice upon subcutaneous immunization with the LaPSA-VLP specifically interacted with the *L. amazonensis* soluble total antigens. LaPSA-VLP-immunized mice elicited significantly greater quantities of parasite-specific IgG from the spleens, popliteal lymph nodes, and footpads than unimmunized mice. LaPSA-VLP immunization also enhanced the proliferation of B cell populations in the spleens of mice and significantly lessened the CL symptoms, notably the footpad swelling and IFN-γ-mediated inflammatory response. Overall, immunizing mice with the LaPSA-VLPs prevented mice from developing severe CL symptoms, signifying their developmental potential.

## 1. Introduction

Leishmaniasis is a parasitic disease caused by more than 20 species belonging to the genus *Leishmania* [1]. Sandflies, especially the species *Phlebotomus* and *Lutzomiya*, are the primary vectors transmitting the disease in the Old and New Worlds, respectively [2]. Cutaneous leishmaniasis (CL) is a disease that is found in both the Old and New Worlds. In the eastern hemisphere, *L. infantum*, *L. major*, and *L. aethiopica* are the common causative agents of CL while species such as *L. braziliensis*, *L. amazonensis*, and *L. mexicana* are the predominant causes in Central and South America [3]. Clinical manifestations of CL vary across species. Characteristic signs associated with CL include the formation of ulcerative skin lesions that have a crustaceous appearance, lymphangitis, and the presence of satellite lesions from parasite dissemination [4]. Old World *Leishmania* species generally inflict self-limiting ulcers, whereas CL caused by some of the New World species such as *L. amazonensis* are capable of transitioning to mucocutaneous leishmaniasis, which results in severe deformities and long-lasting disability [5]. Furthermore, recent findings reported that *L. amazonensis* is also capable of causing visceral leishmaniasis (VL) in both canines and humans, thus highlighting its importance from both clinical and veterinary perspectives [6,7]. Current global CL estimates are reported to be around 12 million people affected with 2 million new cases occurring each year [5]. Given the ongoing climate change crisis, the incidence and geographical distribution of vector-borne diseases such as CL are hypothesized to be affected [8,9]. Drugs are available for CL treatment, but these are not without limitations. For the pentavalent antimonials, they must be administered multiple times and there are also growing concerns regarding drug resistance [10,11]. Other drugs such as miltefosine can be used but their usage is clinically impractical given their high cost and availability issues [4]. Vaccination is considered the most cost-effective method for disease prevention and efficacious vaccines for human CL are currently lacking. For these reasons, there is a pressing need to develop vaccines that can prevent the development of leishmaniasis.

The promastigote surface antigen (PSA) is of particular interest for leishmaniasis vaccine development, as this antigen is expressed in both promastigotes and amastigote stages of various *Leishmania* spp. [12,13,14]. To date, several *L. amazonensis* PSA-based experimental vaccine studies have been conducted against both CL and VL with varying degrees of protection. Recently, the PSA antigen derived from *L. amazonensis* was reported to be capable of conferring protection against canine VL [15,16,17]. Yet, studies assessing the efficacy of PSA vaccines against CL in animals have been predominantly performed using *L. major* or *L. mexicana* challenge infections [18]. To the best of our knowledge, apart from one earlier PSA vaccine study involving a recombinant vaccinia virus vaccine platform [19], CL vaccine studies involving PSA as vaccine antigens paired with *L. amazonensis* challenge infections are severely lacking.

Here, to address this research gap and advance our understanding of PSA vaccine-induced immunity induction against CL, we further evaluated the efficacy of PSA vaccines against *L. amazonensis* infection using the virus-like particle (VLP) vaccine platform. VLPs are nanoparticles that structurally resemble a virus but do not possess the viral genome necessary for replication and are widely utilized as a vaccine platform [20]. VLPs, despite their highly regarded immunogenicity and safety profiles, are underutilized in vaccine studies. To date, only two VLP studies investigating their protective efficacy against VL in vivo have been reported [21,22], while VLP-induced protection against CL remains unexplored. To address this gap in our understanding, we assembled a chimeric influenza VLP expressing the *L. amazonensis* PSA on the surface of the influenza M1 protein. These self-assembled VLPs were acquired by co-expressing the influenza M1 protein along with PSA in insect cells using the baculovirus expression system as demonstrated in our experimental VL vaccine study [22]. Based on our previous VL PSA-VLP study, which reported effective induction of humoral immunity and protection against VL in mice, we hypothesized that a similar phenomenon could be observed against CL as well. Our study revealed that VLPs expressing the *L. amazonensis* PSA is a feasible strategy for effectively controlling CL in animal models.

## 2. Materials and Methods

### 2.1. Mice and Parasites

Six-week-old female BALB/c mice were purchased from NARA Biotech (Seoul, Republic of Korea). All animal experiments were approved and conducted following the guidelines set out by the Kyung-Hee University Institutional Animal Care and Use Committee (Permit ID: KHSASP-21-250). All efforts were made to minimize animal suffering, and animals reaching the humane intervention endpoint were euthanized with CO_2_. Promastigotes of *L. amazonensis* strain LV79 (MPRO/BR/1972/M184) were cultured at 28 °C in M199 complete medium supplemented with 10% heat-inactivated fetal bovine serum (Gibco, Grand Island, NY, USA), 100 units/mL of penicillin–streptomycin (Welgene, LS202-02), 20 mM HEPES (Sigma-Aldrich, St. Louis, MO, USA; H4034), 0.1 mM adenine (Sigma-Aldrich, A2786), 0.0005% hemin (Sigma-Aldrich, 51280), 0.0002% biopterin (Sigma-Aldrich, B2517), 0.0001% biotin (Sigma-Aldrich, B4639), and 4.62 mM NaHCO_3_ (Sigma-Aldrich, S5761). Promastigotes were subcultured every 3 days and underwent no more than four passages.

### 2.2. Generation of Recombinant Baculovirus and VLPs

The codon-optimized LaPSA gene cloned into the pFastBac vector was synthesized by GenScript (Piscataway, NJ, USA). Vectors containing the LaPSA gene were transformed into DH10Bac competent cells. Recombinant LaPSA baculovirus and VLPs expressing the LaPSA antigens were produced following the method previously described [22]. Successful transformation of the LaPSA gene was confirmed by colony PCR using the bacmid DNA extracted from the colonies. Successful transformant bacmid DNA was transfected into Sf9 cells using Cellfectin II (Invitrogen, Carlsbad, CA, USA, 10362100) to produce recombinant baculovirus (rBV) expressing the LaPSA gene. The rBVs expressing LaPSA and influenza M1 were co-transfected into Sf9 cells for VLP assembly. Assembled VLPs were collected from the cell culture supernatants and centrifuged at 6000 rpm, 30 min, 4 °C to remove cell debris. The supernatant fraction containing the LaPSA-VLPs were ultracentrifuged at 30,000 rpm, 30 min, 4 °C, and pelleted fractions were further purified through a 20%–30%–60% discontinuous sucrose gradient using swing bucket rotors at 30,000 rpm, 40 min, 4 °C. Distinct white bands corresponding to the VLP fractions were carefully collected, washed with PBS, and quantified using a MicroBCA protein assay kit following the manufacturer’s instructions (Thermo Fisher Scientific, Waltham, MA, USA).

### 2.3. Confirmation of VLP Assembly, Antigen Expression, and Characterizing the VLPs

The presence of transmembrane domains in the antigen of interest is extremely important for proper influenza VLP assembly [23]. To ensure that transmembrane domains are present within the *L. amazonensis* PSA gene, in silico analysis of this gene was performed using Phobius software (https://phobius.sbc.su.se/, accessed on 7 January 2021; Stockholm Bioinformatics Center, Sweden) as previously described [22]. Western blotting and transmission electron microscopy (TEM) techniques were used to confirm LaPSA expression and successful self-assembly of the VLP vaccines. In brief, LaPSA-VLPs were resolved by SDS–PAGE. Nitrocellulose membranes containing the transferred proteins were blocked with skim milk (5% *w*/*v* prepared in Tris-buffered saline with 0.1% Tween 20, TBST) and incubated at room temperature (RT) for 30 min. Membranes were incubated with *L. amazonensis* polyclonal antibodies or monoclonal M1 antibodies overnight at 4 °C. Membranes were repeatedly washed and allowed to react with horseradish peroxidase (HRP)-conjugated anti-mouse IgG for 1 h at RT. Protein bands were exposed to enhanced chemiluminescence (ECL) solution containing equal volumes of luminol and peroxide and images were visualized using a ChemiDoc Imaging System (Bio-Rad, Hercules, CA, USA). The mean density of each protein band was calculated via ImageJ software (Version 1.54g) (Bio-Rad). For TEM, LaPSA-VLPs adsorbed to grids were stained with 2% uranyl acetate and visualized using a Bio-High voltage EM system (JEOL Ltd. JEM-1400 Plus, Tokyo, Japan) at the Korea Basic Science Institute as previously described [24].

### 2.4. VLP Vaccine Immunization and L. amazonensis Infection in Mice

BALB/c mice (*n* = 4 per group) were randomly selected and subdivided into the Naïve, Naïve+Cha, and vaccination group (SC). LaPSA-VLPs were subcutaneously inoculated into each mouse in the SC group at a dose of 50 μg/mouse. VLPs were administered twice at 4-week intervals. Naïve+Cha and SC groups were subcutaneously infected with 1 × 10^7^ *L. amazonensis* promastigotes through the right footpad at week 8.

### 2.5. L. amazonensis-Specific IgG, IgG1 and IgG2a in Sera, IgG in Spleen, Popliteal Lymph Node (PLN), and Footpad Homogenates

*L. amazonensis*-specific IgG, IgG1, IgG2a antibodies were assessed from the sera acquired after prime and boost immunizations. IgG antibodies were also determined from the spleen, popliteal lymph node (PLN), and footpad homogenates of challenge-infected mice using enzyme-linked immunosorbent assay (ELISA). *L. amazonensis* parasites were sonicated and whole lysate antigens were used as coating antigens for ELISA as described [22]. Briefly, 96-well plates were coated overnight at 4 °C with 5 μg/mL of *L. amazonensis* soluble total antigens in carbonate coating buffer. After washing the wells with phosphate-buffered saline with 0.005% Tween 20 (PBST), the wells were blocked with 0.2% gelatin for 2 h at 37 °C. Next, mouse sera (1:50), spleen supernatant (1:10), PLN supernatant (1:10), and footpad homogenate (1:10) diluted in PBST were prepared and inoculated into the respective wells. Plates were incubated for 1 h at 37 °C, washed with PBST, and incubated for another 1 h at 37 °C with HRP-conjugated secondary antibodies (IgG, IgG1, and IgG2a) purchased from Southern Biotech (Birmingham, AL, USA). Citrate buffer containing H_2_O_2_ and o-phenylenediamine was prepared and 100 μL of this substrate buffer solution was added to each well. After stopping the colorimetric reactions with dilute H_2_SO_4_, optical density readings at 492 nm were measured.

### 2.6. B Cell Responses in PLN by Flow Cytometry

Immune cells from popliteal lymph nodes were prepared for flow cytometric analysis following a previously described method [25]. Briefly, lymphocytes were stimulated with *L. amazonensis* total soluble antigen (5 μg/mL) for 4 h at 37 °C with 5% CO_2_. After antigen stimulation, cells were stained with fluorophore-conjugated IgD (PE) and CD19 (FITC) antibodies purchased from BD Biosciences (558597, 553785). Stained samples were analyzed using an Accuri C6 flow cytometer (BD Biosciences, Franklin Lakes, NJ, USA).

### 2.7. Footpad Thickness, Weight, and IFN-γ

The footpad thickness was measured weekly using a caliper gauge (Kroeplin, Berlin, Germany), and the footpad swelling was calculated as the difference between the thickness of the infected footpad and the thickness of the normal footpad as indicated [26,27]. Mice were sacrificed 10 weeks post-challenge and the weight of the right footpad, which had been infected, was measured. The right footpad was homogenized individually and the supernatants were used to determine inflammatory IFN-γ responses as described previously [24].

### 2.8. Statistical Analysis

All animal samples were prepared on an individual basis and experiments were performed in triplicate. Statistical analysis was performed using GraphPad Prism 8 software (San Diego, CA, USA). Data sets are presented as mean ± SD and significance between the groups was determined using either unpaired Student’s *t*-test or one-way ANOVA with Tukey’s post hoc test. Statistical significance is denoted with an asterisk.

## 3. Results

### 3.1. LaPSA Gene Cloning, VLP Assembly, and VLP Characterization

**Figure 1:**
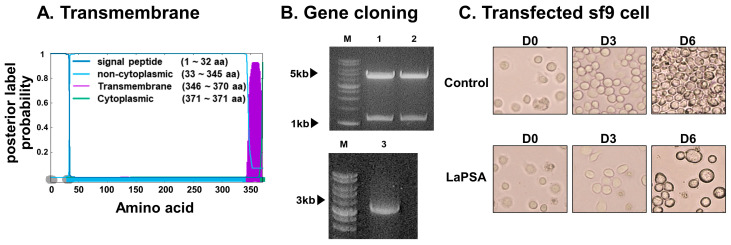
Transmembrane prediction, gene cloning, and assembly of VLPs expressing the *L. amazonensis* PSA. The transmembrane structure of the LaPSA protein was computationally predicted using Phobius, with the amino acid sequence positions plotted on the *x*-axis and their domain occupancy probabilities on the *y*-axis (**A**). *L. amazonensis* gene inserted in pFastBac was transformed and gene expression was confirmed by restriction enzyme digestion (**B**). The upper panel shows the cleaved pFastBac vector LaPSA gene. Colony PCR results depicting the presence of the LaPSA gene isolated from successful transformants are provided in the lower panel. Sf9 cells were transfected with LaPSA bacmid DNA for recombinant baculovirus acquisition, which was later used for VLP assembly (**C**). Images were acquired at 200× magnification.

**Figure 2:**
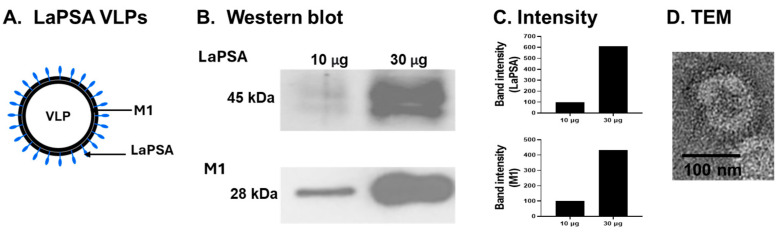
LaPSA-VLP characterization. Schematic diagram depicting influenza virus-like particles expressing the *L. amazonensis* PSA (LaPSA) (**A**). LaPSA-VLPs were characterized by western blotting and transmission electron microscopy. LaPSA and influenza M1 protein contents in the VLPs were detected at 45 kDa and 28 kDa, respectively (**B**). The band intensities of LaPSA and M1 antigens were determined under different protein concentrations (**C**). Morphological features of the LaPSA were confirmed via TEM (**D**). Dark bands near the spherical surface, indicating LaPSA expression, were observed.

The transmembrane topology and signal peptides were predicted utilizing the Phobius web server (Figure 1A). This software predicts the likelihood of each amino acid’s position within various regions or domains, with the x-axis indicating the amino acid position and the y-axis representing the probability associated with each respective position. Codon-optimized LaPSA was inserted into the pFastbac1 plasmid. Restriction enzyme digestion with BamHI and XhoI confirmed the integration of the 1116 bp LaPSA gene (Figure 1B, lanes 1 and 2). Successful LaPSA gene cloning was further confirmed by colony PCR using the bacmid DNA acquired from transformants (Figure 1B, lane 3). LaPSA-rBV was generated by transfecting the transformant bacmid DNA into Sf9 cells (Figure 1C). Sf9 cells were monitored for 6 days and changes in cell viability and morphology were observed. In the untransfected control, aberrant cellular enlargement or lysis indicative of transfection was not detected. Rather, cells continued to divide and eventually became overconfluent by day 6. Contrastingly, Sf9 cells transfected with the LaPSA bacmid DNA underwent noticeable changes. Transfected cells never reached confluence and much of each transfected cell became enlarged as displayed by the images acquired 6 days post-transfection. A graphical illustration showing the general structure composition of the LaPSA-VLP is provided (Figure 2A). Successfully self-assembled VLPs were characterized by western blotting and TEM (Figure S1). The expression of both LaPSA and the core influenza M1 antigen were detected at 45 and 28 kDa, respectively, and densitometric analyses corresponding to these bands were also provided (Figure 2B,C). TEM image revealed that the LaPSA-VLPs were properly assembled, as indicated by the presence of a darkened region near the surface indicative of LaPSA gene expression (Figure 2D).

### 3.2. Animal Experimental Schedule

**Figure 3:**
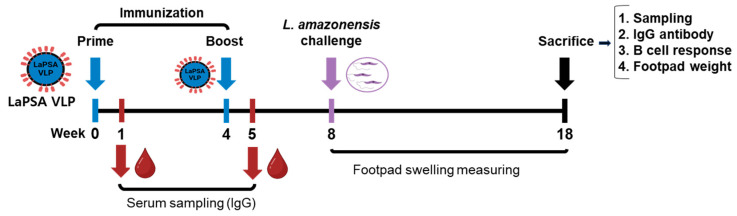
Animal experimental design. All experiments involving animals were conducted following the schedule depicted in the schematic diagram. BALB/c mice were subcutaneously immunized with LaPSA-VLPs with blood collection at regular intervals. Four weeks after the final immunization, mice were challenged with *L. amazonensis*, and various immunological assays were performed to assess vaccine efficacy.

Mice were immunized following the experimental schedule illustrated in Figure 3. Briefly, two subcutaneous immunizations with the LaPSA-VLPs at 4-week intervals were performed and blood collection was performed 1 week after each immunization. Four weeks after the final immunization, mice were challenged with *L. amazonensis* promastigotes. The disease progression was carefully checked for 10 weeks, during which the thickness of the plantar lesions was measured weekly. At the end of this monitoring period, the mice were sacrificed and individual samples were collected for immunological assays.

### 3.3. IgG Antibody Response in Sera, Spleen, PLN, and Footpad

**Figure 4:**
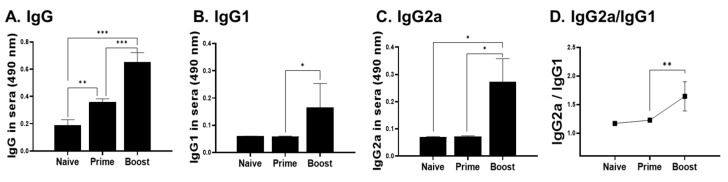
Parasite-specific antibody response detection. ELISA was performed to assess *L. amazonensis*-specific IgG responses using the sera of immunized mice. Sera were acquired after each immunization. Data are presented as mean ± SD (* *p* < 0.05, ** *p* < 0.01, *** *p* < 0.001) from experiments performed in triplicate and statistical significance was determined using ANOVA.

**Figure 5:**
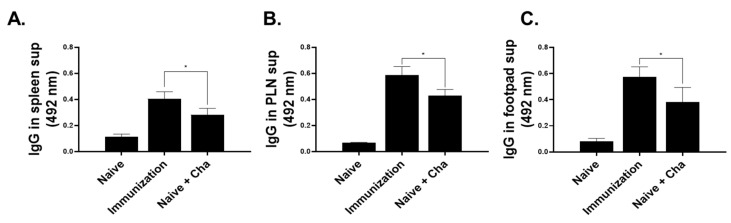
*Leishmania*-specific IgG acquired from the spleen, PLN, and footpads. Mice were sacrificed and organs were sampled to assess parasite-specific IgG responses. ELISA was performed using homogenates acquired from spleen (**A**), PLN (**B**), and footpads (**C**). Data are presented as mean ± SD (* *p* < 0.05) from experiments performed in triplicate and statistical significance was determined using ANOVA.

The right footpads of BALB/c mice were subcutaneously immunized with LaPSA-VLPs and sera were collected 1 week after each immunization. Sera acquired after priming showed significantly higher levels of an *L. amazonensis*-specific IgG antibody response compared to naive controls. Enhancements of the antibody response induction (IgG, IgG1, and IgG2a) became more pronounced upon boost immunization (Figure 4A–C). An IgG2a-dominant response was found after boost but not upon prime immunization (Figure 4D). Four weeks after the final immunization, mice were challenged with 1 × 10^7^ *L. amazonensis* promastigotes and *L. amazonensis*-specific antibody responses were evaluated from the spleens, PLN, and footpads of mice. Subcutaneous immunization of LaPSA-VLP elicited significantly increased parasite-specific antibodies in the spleens of mice compared to Naïve+Cha controls (Figure 5A). Identical findings were also observed in the PLN (Figure 5B) and the footpads of mice (Figure 5C), indicating that LaPSA-VLPs are an effective inducer of humoral immunity.

### 3.4. B Cell Response in the PLN

**Figure 6:**
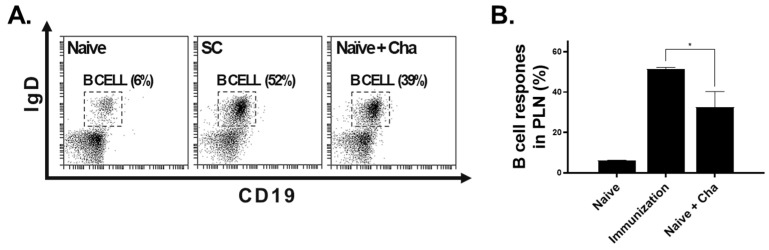
Flow cytometric assessment of vaccine-induced B cell responses. Mice were sacrificed and splenocytes were isolated for flow cytometry. Splenocytes were stained with appropriate surface antibodies and B cell populations were analyzed. B cell responses were determined (**A**) and representative scatter plots for each group were provided (**B**). Data are presented as mean ± SD (* *p* < 0.05) and statistical significance was determined using ANOVA.

To further assess the humoral immunity elicited by the VLP vaccines, B cell proliferation in the lymph nodes was assessed by flow cytometry. Total B cell responses were assessed by staining lymph node cells with B cell-specific surface antibodies (Figure 6A,B). Consistent with the ELISA data, LaPSA-VLP immunization resulted in a substantial increase in B cell responses compared to the Naïve+Cha control.

### 3.5. LaPSA-VLP Immunization Lessened the Severity of CL and Inflammation

**Figure 7:**
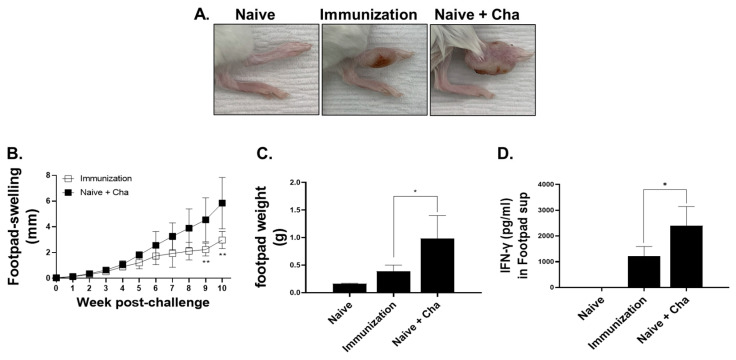
Footpad swelling and weight changes following *L. amazonensis* infection. *L. amazonensis* promastigotes were subcutaneously inoculated into the footpads of mice and pathological changes were monitored. Representative images for naïve (Naive), LaPSA-VLP immunized (Immunization), and unimmunized infection control (Naive+Cha) are shown (**A**). Images were acquired at week 10 post-challenge infection. Mice were infected with *L. amazonensis* promastigotes and the footpad thickness of individual mice was recorded. Footpad swellings were observed in infected mice and changes were recorded on a weekly basis (**B**). Data are presented as mean ± SD (** *p* < 0.01), and statistical significance was determined using Student’s *t*-test. On week 10 post-challenge, the mice were sacrificed, and the footpads were weighed (**C**). IFN-γ cytokine production was measured from the footpads of the mice (**D**). Data are presented as mean ± SD (* *p* < 0.05) and statistical significance was determined using ANOVA.

To confirm the effect of our VLP vaccines in immunized mice, the right footpads of the mice were carefully monitored after challenge infection. As expected, cutaneous lesions or footpad thickening was not detected in the naïve control group (Figure 7A). However, thickened footpads were observed in both the LaPSA-VLP and Naïve+Cha groups (Figure 7A). These lesions, however, were less pronounced in the LaPSA-VLP immunization group compared to the Naïve+Cha group. Specifically, footpad swelling and cutaneous lesions were less severe in the immunization group, indicating that vaccination diminished the degree of tissue inflammation elicited by cutaneous leishmaniasis. Contralateral footpads of VLP-immunized and Naïve+Cha mice were measured weekly for 10 weeks following the challenge infection. For the first 4 weeks post-infection, footpad thickness was similar for the two groups (Figure 7B). Differences in footpad swelling became noticeable from week 5 onwards, with less severe swelling being detected in the LaPSA-VLP group. However, the means between the two groups at these time points were not statistically significant until week 8 post-challenge. A significant reduction in footpad thickness was detected in the LaPSA-VLP group throughout the remaining weeks, indicating vaccine-induced alleviation of CL immunopathology. The footpads of all mice were weighed after euthanasia. *L. amazonensis* challenge infection incurred footpad thickening regardless of immunization, but the weight of the footpads was much heavier in the Naïve+Cha than in the VLP group (Figure 7C). Overall, the footpad weight for the LaPSA-VLP group was comparable to that of the naïve, and significantly less than that of the Naïve+Cha control. The concentration of IFN-γ was also evaluated from the footpad supernatants of mice (Figure 7D). LaPSA-VLP immunization significantly reduced the production of IFN-γ in mice compared to the unimmunized control mice.

## 4. Discussion

In this study, LaPSA-VLP vaccines were generated and the protective immunity induced by LaPSA-VLP vaccines was evaluated in mice. LaPSA-VLP immunization induced humoral immune responses, as indicated by the enhanced parasite-specific IgG antibody responses in the sera, spleens, PLN, and footpad homogenates. The LaPSA-VLPs also enhanced the splenic B cell response post-challenge infection. These immune responses evoked through LaPSA-VLP immunization contributed significantly to reducing the severity of CL upon challenge infection.

To date, various vaccine platforms have used numerous *Leishmania* spp. antigens to develop an efficacious CL vaccine, which include but are not limited to recombinant subunit vaccines, DNA vaccines, live-attenuated pathogens, or computationally identified epitopes. Nonetheless, findings from a vast majority of the CL vaccine studies reported to date were conflicting as efficacies against CL in animal models greatly varied. Bacteria-derived recombinant PSA2 of *L. major* elicited Th1 immunity in mice, but the vaccine failed to induce protective immunity against CL [28]. Similarly, immunizing BALB/c mice with the *L. infantum* ribosomal P0 protein failed to prevent CL progression [29]. On the contrary, other CL vaccines tested in rhesus macaques were proven to be efficacious as demonstrated by the absence of lesion formation [30]. Later studies revealed that the efficacy of these subunit vaccines could be further optimized by pairing with appropriate adjuvants [31]. These conflicting findings were also reported from DNA vaccines as one study reported full protection against *L. major* using DNA vaccines, while only partial protection was observed in others [32,33]. While live parasites can be efficacious, their usage is strongly discouraged for various reasons [34,35]. Other studies also reported that heterologous immunization regimens involving DNA and viral-vectored vaccines enhanced protection [36,37,38]. The potential of multi-epitope CL vaccines using bioinformatics was also reported [39,40]. So far, no one has attempted to use VLPs as a vaccine platform for CL vaccine development. Th1 immunity is a well-documented inducer of protection against CL [41,42,43]. Among the cytokines associated with Th1 immunity, IFN-γ is of particular interest for its crucial role in intracellular parasite clearance [44]. In our study, *L. amazonensis* PSA-VLP vaccines elicited a high IgG2a/IgG1 ratio after boost immunization, suggesting a Th1-bias is necessary for parasite clearance. As a result of this Th1-mediated parasite clearance, footpad swelling was observed to a lesser extent in vaccinated mice, which correlates with their diminished quantities of IFN-γ.

VLPs are potent inducers of humoral immunity, partly due to their small size allowing easy trafficking to the lymph nodes for naïve B cell activation [45]. Evidently, in the present study, robust B cell induction was observed after LaPSA-VLP immunization, which far surpassed those of the Naïve and Naïve+Cha groups. Consistent with this study, our PSA-VLP involving VL also elicited strong B cell, germinal center B cell, and memory B cell responses in the spleens of immunized mice [22]. While VLPs generally do not require adjuvants, their immunogenicity could be further enhanced through adjuvant incorporation. Multiple studies have documented that vaccine efficacy can be potentiated using various adjuvants, including alum [46], CpG-ODN [47,48], and others [49,50]. Though testing the effect of these adjuvants was beyond the scope of this study, their role in enhancing VLP-mediated vaccine efficacy is worth investigating and should be performed in the future.

The challenge infection dose for a pathogen is a critical factor that can influence vaccine efficacy. Strong correlations can be drawn between infection dose and disease severity, as mice infected with a high infection dose succumb to death while those affected by low amounts of pathogens do not follow the same fate. In line with this notion, mice infected with 10LD_50_ of influenza A virus were not protected whereas mice receiving the 2LD_50_ infection inoculum were better protected [51]. In the present study, mice were challenge-infected with a high dose of *L. amazonensis* promastigotes (1 × 10^7^). Compared to earlier studies [26], which used 10^3^, 10^4^, or 10^5^ promastigotes as the infection inoculum, the dose used in the present study was at least 10^2^- or 10^3^-fold greater and the overall findings are encouraging. The footpad swelling in immunized mice from week 9 onwards was significantly reduced and the overall weight of the footpads was significantly lower than those of unimmunized infection control mice. Consistent with an earlier study reporting PSA-mediated protection against *L. amazonensis* [19], similar findings were also observed in our study even with the differences in challenge infection dose.

Recent findings reported that intranasally administering *L. amazonensis* promastigote antigen enhanced host resistance to *L. amazonensis* infection in BALB/c mice, whereas parenteral routes such as subcutaneous injections failed to do so [52]. Similar findings were also observed in intranasally immunized C57BL/6 mice [53]. In contrast to intranasal delivery, parenteral routes were found to be ineffective and were associated with enhanced disease susceptibility [54,55]. Consistent with this finding, phase 3 clinical trial results involving killed *L. amazonensis* immunization through intramuscular route offered no protection against CL [56]. In our present study, subcutaneous immunization significantly lessened the footpad swelling and weight compared to unimmunized controls. Given these findings, alternative routes that ensure mucosal immunity induction such as intranasal or oral vaccines should be explored to overcome these limitations and enhance vaccine efficacy.

There are two major limitations to this study. First, this study did not assess the actual protection elicited by the VLP vaccines, either by limiting dilution assay or via PCR. While footpad swellings were significantly reduced, this does not necessarily indicate reductions in footpad parasite loads. Another limitation of our study is the inherent issue of inoculating *L. amazonensis* through the footpads, which can be simultaneously advantageous and disadvantageous [57]. In general, the footpad lesions do correlate with the parasite burden present within the local site of infection. However, once ulcerations in footpads become noticeable, as was the case in our study, parasitic dissemination into the visceral organs is possible [58]. Consequently, the parasite burden detected from this localized lesion may not be an actual representation of the true parasitic load. Furthermore, an earlier study reported that footpad swelling can occur following challenge infection without a substantial increase in the parasite burden [59]. These aspects should be considered for all future CL vaccine studies to accurately assess vaccine-mediated protection.

## 5. Conclusions

This study aimed to address the limitations of conventional vaccine platforms by developing a VLP vaccine expressing the PSA of *L. amazonensis*. Our findings demonstrated that LaPSA-VLP immunization elicited parasite-specific IgG antibody responses in sera, spleen, PLN, and footpads that contributed to lessening the severity of CL incurred by *L. amazonensis* infection in BALB/c mice. The findings presented here underscore the potential of the LaPSA-VLPs as a CL vaccine candidate and should be the focus of further study.

## Data Availability

Data supporting the findings of this study are contained within the article.

## Supplementary Materials

The following supporting information can be downloaded at: https://www.mdpi.com/article/10.3390/vaccines12070793/s1, Figure S1: The whole western blots for LaPSA (A) and influenza M1 protein (B).
