# Supplementary figures and images for: Immunizing Mice with Influenza Virus-like Particles Expressing the Leishmania amazonensis Promastigote Surface Antigen Alleviates Inflammation in Footpad
Source: Vaccines (Basel). 2024 Jul 18;12(7):793. doi: 10.3390/vaccines12070793 (PMC11281337; doi:10.3390/vaccines12070793)

## Slide 1
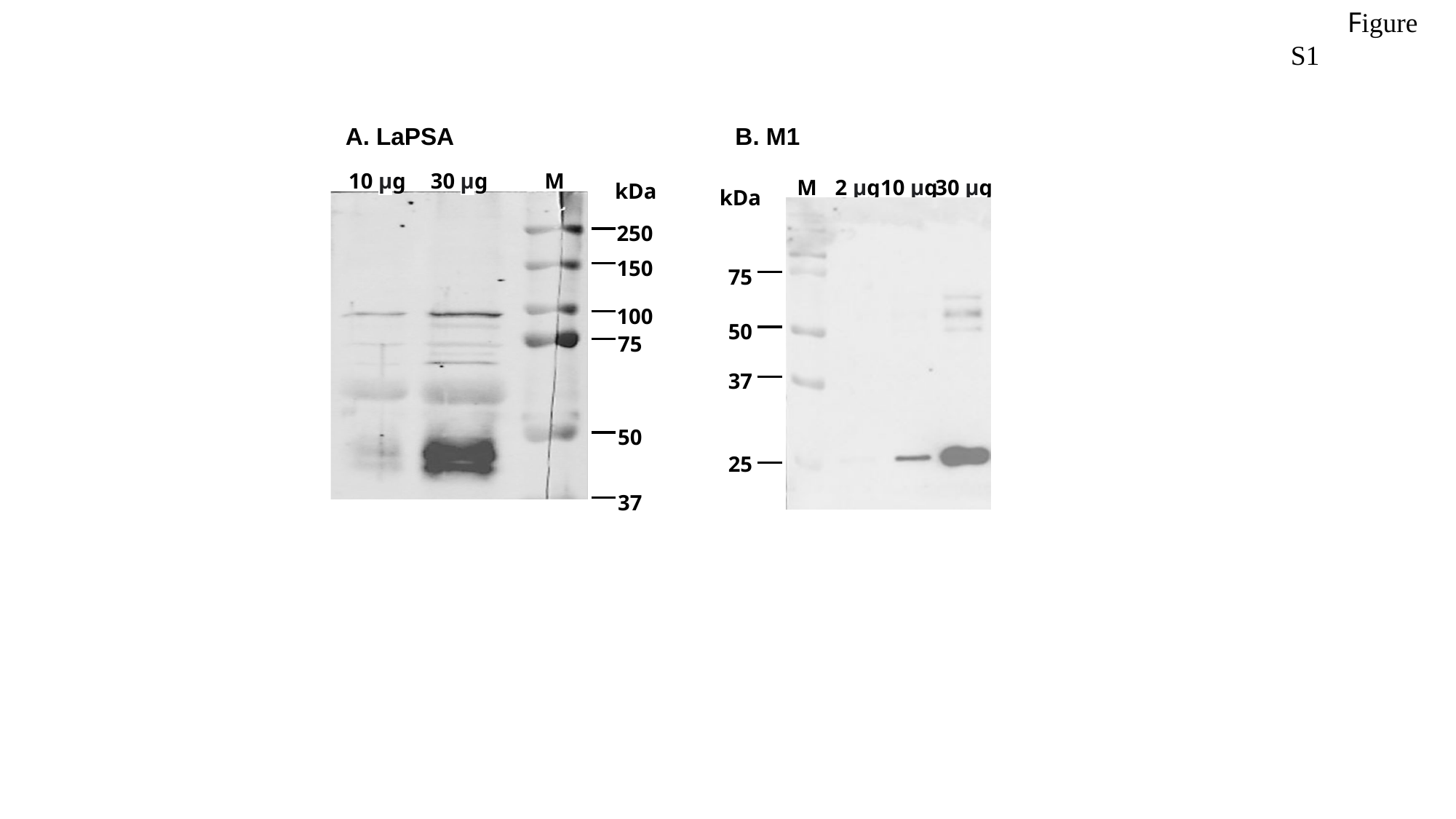

Figure S1
 A. LaPSA B. M1
10 μg
30 μg
M
kDa
250
150
100
75
50
37
M
2 μg
10 μg
30 μg
kDa
75
50
37
25

Supplement: Supplementary file 1 [file vaccines-12-00793-s001.zip › vaccines-3071695-supplementary.pptx]
